# Ecological Factors Influence Diversity, Structure, and Regeneration Status of Woody Plant Species in Basso Subwatershed, Gamo Zone, Ethiopia

**DOI:** 10.1155/sci5/8890087

**Published:** 2025-10-13

**Authors:** Wondwesen Tefera, Wakshum Shiferaw, Gezahegn Kassa

**Affiliations:** Department of Natural Resource Management, College of Agricultural Sciences, Arba Minch University, P. O. Box 21, Arba Minch, Ethiopia

**Keywords:** Basso Subwatershed, CCA, diversity, environmental factors, regeneration status, woody plant species

## Abstract

Ethiopian woodlands offer a variety of cultural, social, and economic advantages. However, human disturbances, climate change, and changes in land use may have influences on the benefits and services that woodlands currently provide. Furthermore, the distribution of plant ecosystems can be influenced by ecological characteristics. This study aimed to identify the types of plant communities, the relationships between plant communities and environmental conditions, the diversity, composition, and structure of woody plant species, and the regeneration status of woody species in the Basso Subwatershed. To gather information on vegetation and environmental conditions, systematic random sampling was employed. Fifty-two 20 m × 20 m sample quadrats were set up to assess trees and shrubs, and five 5 × 5 m subplots were created to assess seedlings and saplings. Woody species diversity was evaluated using the Shannon–Weiner diversity index, species richness, and Shannon evenness. Canonical correspondence analysis was used to examine links between vegetation and the environmental factors. In this study, a total of 28 plant species that belong to 17 families and five different plant community types were identified. The richness and diversity of species differed depending on the type of community. The overall Shannon–Wiener diversity index (2.65), species richness (25), and the Shannon evenness value (0.84) of the woodland were recorded in the subwatershed. Findings show that slope, disturbance, and grazing intensity reflected significant effects on woody vegetation patterns in the Basso Subwatershed (*p* < 0.01). However, altitude, aspect, and human impacts did not show significant effects on woody vegetation patterns in the Basso Subwatershed (*p* > 0.01). The most dominant woody species in the subwatershed were *Acalypha fruticosa*, *Terminalia brownii*, *Combretum molle*, and *Balanites aegyptiaca*. In the future, rehabilitation and sustainable vegetation management techniques will take into consideration significant environmental factors, particularly for species with low rates of regeneration like *Dichrostachys cinerea*, *Grewia bicolor*, and *Maytenus senegalensis.*

## 1. Introduction

Woodlands account for 14% of Africa's total area and more than 40% of all tropical forest zones on Earth [1]. Woodlands provide a wide range of economic, social, and ecological benefits, from cultural values to tangible economic rewards [2]. In eastern Africa, for example, fuel wood serves as the main energy source, and timber is extensively utilized in the building sector [3]. Furthermore, this significant capital asset can be vital to ecosystem health, human life, and general functioning if properly managed. However, overgrazing, deforestation, irresponsible wood harvesting practices, ecosystem degradation, and changes in land use are some of the human-caused issues that have deteriorated woodlands. The capacity of woodlands to deliver rich ecosystem services has been severely damaged by various socioeconomic and environmental issues that have resulted from these variables [4]. The capacity of woodlands to provide vital ecosystem services may also be negatively impacted by climate change, which could exacerbate the stresses [5]. According to IPC (2007), the assessment report (AR4) states that climate change may have a substantial influence on several aspects of biological diversity [6]. Moreover, ecological features can affect how plant habitats are distributed [7]. These will impact ecological interactions, genetic diversity within species, species that make up ecosystems, and ecosystems themselves. The composition and stand structure of plant communities, biodiversity, biogeochemical cycles, and ecosystem physical features are all significantly impacted by changes in land cover. They affect climate regulation as well [8, 9]. These changes are caused by a variety of natural and human forces [10]. The effects of natural vegetation like changes in temperature take years to manifest themselves, whereas the effects of human action can be immediate and even severe. Land use land cover changes (LULCC) have been connected to notable negative impacts on ecosystems that have been observed locally, regionally, and internationally. High levels of pollution in the air, water, and soil could arise from the observed LULCC. Biodiversity is reduced when land that has primarily been left undisturbed is put to more intensive uses, including farming, grazing cattle, and selective tree harvesting [11]. Tropical deforestation was the main factor responsible for the average annual CO_2_ emissions in the 1990s, which varied between 0.5 and 2.7 GtC [12]. These modifications of vegetation impacted the capacity of biological systems to meet human needs, modify ecosystem services, and partially predict how vulnerable individuals and locations are to sociopolitical, economic, and climatic shocks [13].

The need of land for crop cultivation, grazing, and fuel wood production is also the main cause of forest and woodland clearing in Africa [14]. Among the sub-Saharan African nations experiencing these challenges are the Ethiopian people; similar to the case with most developing countries, the primary human factor is population growth [15, 16]. A vast range of vegetation resources, including plantations, high forests, woodlands, bushland, and trees outside of forests, can be found in Ethiopia [17]. Woody Biomass Inventory and Strategic Planning Project (WBISPP) [18] states that the land area of the nation is split into three categories: 29.24 million hectares of woodland (25.5%), 26.4 million hectares of shrub land (23.1%), and nearly 4 million hectares of high forest (3.56%) of the total area. Woodland vegetation of Ethiopia offers many ecological and socioeconomic benefits [14]. In addition to forest resources, the preservation of woodlands can guarantee the availability of abundant biological resources [19].

The restorations of woodland areas of Ethiopia involve a range of stakeholders and strategies to restore the woodlands. Governmental and nongovernmental organizations, as well as the commercial sector, are the primary stakeholders for the restoration of woodlands. Common restoration techniques used today include reforestation, afforestation, and restoration of forests and lands in arid areas accomplished through smallholder tree plantations on farmlands, buffer zone plantations of surrounding natural forests, and agroforestry techniques on farmlands. These strategies are widely employed in the Amhara National Regional State, Tigray National Regional State, South Ethiopia Regional Government, and Oromia National Regional State [20]. Nonetheless, a decrease in coverage and ongoing threats from various extractive human activities are effects on woodlands [21].

The Basso Subwatershed woodland in the South Ethiopia Regional Government provides the majority of the surrounding villages with pasture, firewood, building materials, medical supplies, honey production, and tomato stakes. Additionally, it provides habitat for a variety of fauna. However, little research was done on plant diversity, woody species structure, and regeneration status of woodland [22]. A sustainable use of vegetation is possible with an adequate understanding of the dynamics of plant species regeneration and the variables influencing important tree species [23]. Despite the paucity of research in the studied locations, this information may be essential for integrating restoration initiatives with long-term management plans. Moreover, there was a clear gap that woody plant composition and diversity did not associate with environmental factors in the existing literature. In this study, species composition and diversity of woody plants were examined, types of plant communities were identified, the regeneration status of woody plant species was examined, and the interaction between plant communities and certain environmental conditions in the Basso Subwatershed was examined. The following research questions are addressed in this study: How diverse and what types of woody plants are there in the research area? In the research area, how many types of plant communities were identified? What appears to be the connection between environmental factors and plant communities? What are the regeneration states of woody plant species in the woodland?

## 2. Materials and Methods

### 2.1. Description of the Study Area

Gamo Zone is one of the administrative zones in the South Ethiopia Regional Government. The administrative center of the zone is Arba Minch. Arba Minch is located at 505 km South of Addis Ababa and 120 km South of Wolaita Sodo town. The highest place in this zone is Mount Gughe which is 4207 m a.s.l. Gamo Zone is bordered on the south by the Derashe and Amaro special woredas, on the southwest by South Omo, on the west by Gofa Zone, on the north by Wolaita Zone, and on the northeast by Oromia Regional State [24].

**Figure 1:**
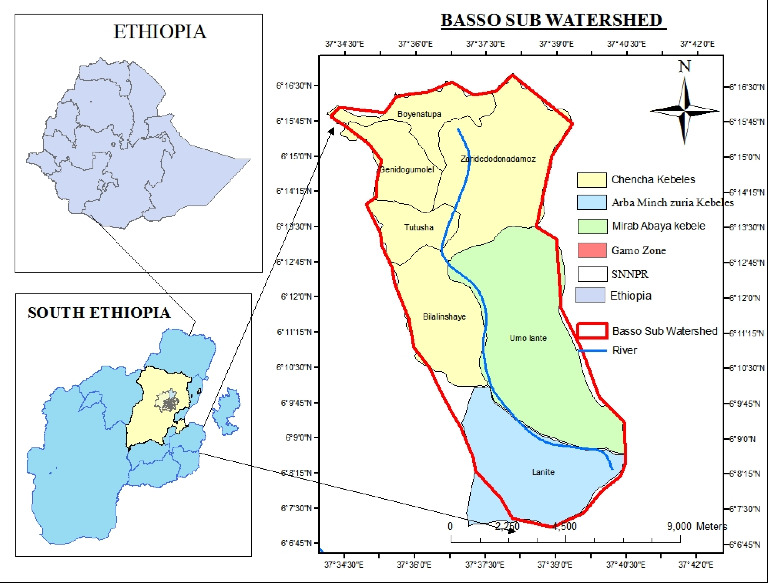
Map of the study area.

The Basso Subwatershed is located in the area between Chencha, Mirab Abaya Woreda, and Arba Minch Zuria. The main route from Addis Ababa to Arba Minch crosses the lower portion of the study watershed. The research location is located between latitudes 606′45″ and 6016′30″ north and longitudes 37°34′30″ and 37°40′30″ east (Figure 1). It covers 10,684 ha. According to a personal GPS reading, the study area's elevation ranges from 1180 to 1850 m.a.s.l.

**Figure 2:**
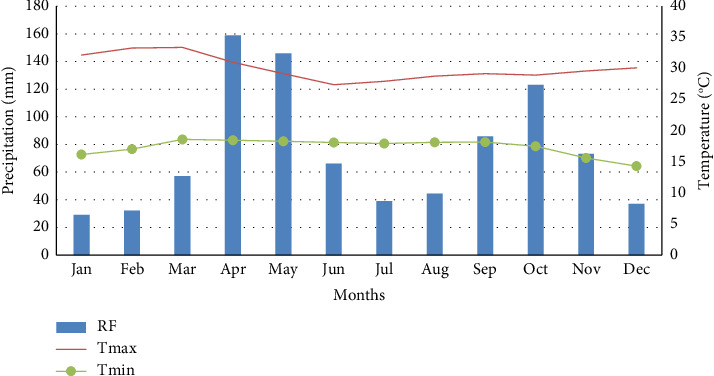
The mean monthly rainfall, minimum, and maximum temperature of the study area.

Rainfall and temperature data for the Basso Subwatershed were obtained from the nearest Arba Minch University Meteorology Service Center. The rainfall distribution is bimodal; the driest months were December and January, with 29.2 mm of rainfall; the first greatest monthly rainfall, 158.9 mm, happened from April to May; the second peak, 123 mm, occurred from September to November (Figure 2), and thus, the annual rainfall ranged in the study area is 29.2–892.4 mm. Monthly maximum temperatures typically fall between 27.40°C and 33.40°C. From 1987 to 2020, the mean monthly minimum temperature was expected to be between 14.3°C and 18.6°C. The average annual temperature was 30.1°C, and the average rainfall was 892.4 mm (Figure 2).

The dominant soil types in the study areas were Vertisols, Nitisols, Leptosols, and Luvisols, with smaller portions of Fluvisols and Cambisols. Based on the data obtained from CSA [25] population projection from (2010–2014), the kebeles in the subwatershed had a projected total population size of 9572, of which females accounted for about 4886 and about 4686 were males. The economy of the study area was predominantly agricultural. According to the Gamo Zone Agricultural Department (GZAD) [26], mixed crop cultivation and livestock rearing were the main agricultural activities of the population of the study areas. Different types of crops were cultivated by the farmers. The highland was moderately productive; wheat, barley, and pulses were dominant crops grown in this area. The farmers earned their income from sales of crops, livestock, and fodder. Mid-altitude was moderately productive and main crops grown in this agroecology zone are maize, sorghum, teff, pulses, wheat, and oil seeds. Households also kept livestock (cattle and goats). Lowland altitude was dominated by agropastoralist livelihood strategies. Main crops are sorghum (*Sorghum bicolor* L. Moench), teff (*Eragrostis tef* L.), maize (*Zea mays* L.), and cassava (*Manihot esculenta* Crantz), and the most common fruits produced in the zone including the study area are mango, banana, avocado, and papaya, while livestock is composed of cattle and goats. The estimated total livestock population number of cattle, sheep, goats, chickens, and equine was 60,825, 37,014, 18,260, 63,864, and 8645, respectively [26].

### 2.2. Site Selection

A reconnaissance survey of the study area was undertaken in July 2022 to get an overview of observations about the area and to determine the sampling method, transect orientation, and number of sample sites to be used for vegetation data collection. The survey focused on identifying the vegetation physiognomy (growth forms: tree or shrub, saplings, and seedlings), determining representative sampling sites, and becoming familiar with the study area. Thus, during the reconnaissance survey, the sapling method and orientation of transects were determined.

### 2.3. Sampling Design and Data Collection

To investigate plant species composition, plant species population structure, and regeneration status of woodland vegetation of Basso watershed, a field survey using systematic random sampling techniques was used. Nine transect lines oriented in the southwest to northeast direction at a distance of 300 m were laid out. Quadrat sizes for trees, shrubs, saplings, and seedlings vary frequently in tropical vegetation studies; nonetheless, nested quadrats are a popular method. Trees and shrubs are usually sampled in bigger quadrats (20 × 20 m), while saplings and seedlings are sampled in smaller nested quadrats (5 × 5 m or even 1 × 1 m) inside the larger quadrat [27]. Hence, for this study, a total of fifty-two sample quadrats with a size of 20 × 20 m (400 m^2^) were established at each transect line at 50-m intervals. This interval was set based on the rise and fall of the altitudes or slopes in the woodland landscape. In each main quadrat, five subplots with a size of 5 × 5 m (25 m^2^) were established, four at each corner and one at the center, to collect data for seedlings and saplings [28, 29]. For each quadrat, physiographic factors such as altitude, aspect, and slope were recorded. Following Kent and Coker [30], data for trees, shrubs, and woody climbers diversity were collected. The Shannon–Wiener diversity index, species richness, and Shannon evenness were used to determine plant species diversity. In each quadrat, all woody plant species were measured, counted, identified using vernacular names, and documented. Despite this, plant species cover abundances in each quadrat were utilized as inputs for analysis of plant species diversity [31].

The dates of the vegetation data collection were July 13, 2022, through August 3, 2022. Individual plants of all trees and shrub species with diameter at breast height (DBH) ≥ 2.5 cm were counted and their diameter recorded in each of the major sample quadrats (400 m^2^). Height of individual trees and shrubs ≥ 2 m was recorded for every woody individual plant having DBH ≥ 2.5 cm [32]. A caliper was used to measure diameter, while clinometers were used to measure slope. A calibrated stick was used to measure tree heights in the study areas. Trees with multiple stems arising from the ground level were measured individually and developed a common DBH of all stems by summing up their square roots following [33].

Saplings are young woody plants with a DBH of less than 2.5 cm and a height of more than 1 m and less than 2 m, whereas seedlings are woody plants with a DBH of less than 2.5 cm and a height of less than 1 m [34]. The cover abundance values of each species were estimated in percentage and converted to 1–9 scales following the procedure of modified Braun–Blanquet scales by Maarel [35]. Plant species were identified by writing down the local names of woody species after requesting local experts and wise elders for their assistance. Identification of scientific names of plant species was carried out in the field and office. Specimens of identified and unidentified species were collected, pressed, and dried properly, following standard herbarium procedures. After vernacular names are known, the scientific names were identified with the help of flora of Ethiopia and Eritrea and the scientific names were also identified using [36] and online websites of the World Flora Plant Net software.

### 2.4. Physiographic and Anthropogenic Variables

In each sampling quadrat, physiographical variables such as altitude and geographical coordinates were measured using Garmin 72 GPS. Aspect was determined using compass. As a possible indicator of total solar energy, the aspect was coded following Ademe et al. [37] and Temesgen and Warkineh [29]. Thus, north = 0; east = 2; south = 4; west = 2.5; northeast = 1; southeast = 3; southwest = 3.3, and northwest = 1.3 [38]. Grazing intensity was estimated based on visual observation of different symptoms of livestock effects such as dung droppings and herbage cuttings following scales designed by [39]. The degree of human interference in each quadrat was estimated as follows. A 0–3 subjective scale was used to record the presence or absence of stumps, logs, and signs of fuel wood collection. The magnitude of the impacts was quantified following 0 = nil; 1 = low; 2 = moderate; and 3 = heavy [40].

### 2.5. Data Analysis

#### 2.5.1. Diversity Analysis

Woody species diversity in the subwatershed was estimated using Shannon–Wiener diversity index (H′) as follows [36].

Shannon–Wiener diversity index (H′) is calculated as follows:

(1) $$\begin{array}{c} H^{\prime }=-\overset{s}{\underset{i=1}{\sum }}P_{i}\;\ln\;P_{i}, \end{array}$$

where *p* is the proportion (*n/N*), where individuals of one particular species found (*n*) divided by the total number of individuals found (*N*), ln is the natural log, Σ is the sum of the calculations, and *s* is the number of species.

Shannon's equitability (*E*) or evenness is determined by the following equation:

(2) $$\begin{array}{c} E=\frac{H^{\prime }}{H_{\max}}=\frac{H^{\prime }}{\ln_{s}\;}, \end{array}$$

where *S* is the number of species recorded.

#### 2.5.2. Similarity Analysis

Sorensen's similarity coefficient (SSC) was computed to determine the patterns of species turnover among successive communities. To find differences between woody plant species among community types in the woodland, the SSC was calculated. The SSC result showed a number between 0 and 1, indicating that total community overlap was equal to 1, and complete community difference was equal to 0. It is used to measure similarities between two habitats and described using the following formula [30]:

Sorensen similarity coefficient:

(3) $$\begin{array}{c} \text{Ss}=\frac{2a}{2a+b+c\;}, \end{array}$$

where Ss = Soreness's similarity coefficient, *a* = number of species common to both samples, *b* = number of species in sample 1, and *c* = number of species in Sample 2.

### 2.6. Classification of Plant Community

**Figure 3:**
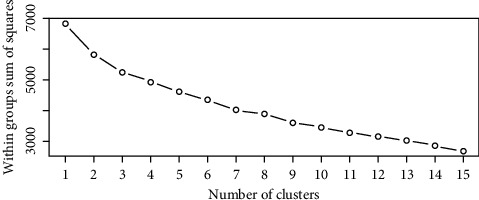
Optimum number of cluster or number of community types for woodlands of Basso Subwatershed.

**Figure 4:**
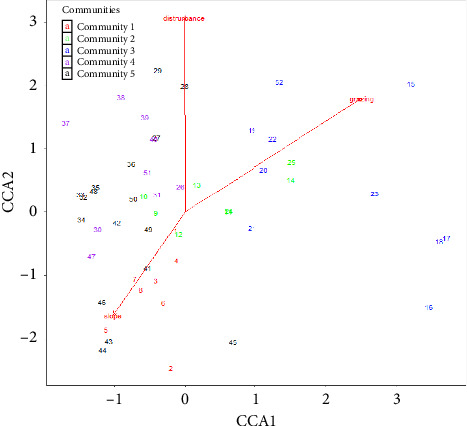
In CCA, sites with plant species are controlled by some environmental variables.

Using the open-source statistical program [38], hierarchical cluster analysis was used using similarity ratio (SR) with colored leaves and labels to categorize different community kinds. The analysis was based on the synoptic abundance value of the species. Synoptic cover abundance value of each species was determined using R-statistical software package. The package for determining the optimal number of clusters was used to decide the number of plant community types (Figure 3). Ward's method and Euclidean distance or SR were used to draw the Dendrogram showing the linkage among the five clusters (Figure 4). Community names were given after one or two species that had higher mean indicator values. In all observed plant communities analysis, species with higher indicator values those that were easily observed repeating themselves in associations. Dominant species are defined as the most distinguishing species of each group, and present in the majority of quadrats belonging to that group [33].

### 2.7. Analysis of Vegetation Structure

**Figure 5:**
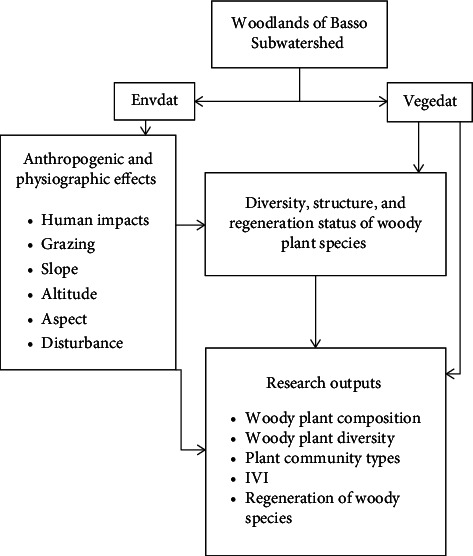
Conceptual framework of the study.

The structures of the vegetation were analyzed by computing diameter at frequency, density, DBH, height, basal area, and importance value index (IVI) (Figure 5). The DBH and tree height were classified into DBH classes and height classes, respectively. The frequency distribution of individuals in each class was calculated. The tree or shrub density, basal area, and values were computed on hectare basis. These vegetation data were computed and summarized using Microsoft Office Excel spread. IVI is a unitless score calculated by adding the relative dominance (RDO), relative density (RD), and relative frequency (RF) of a species [30, 41]. Comparing the ecological value of a species is helpful and an excellent index for determining vegetative features and prioritizing species for management and conservation strategies [42].

The basal area per tree is calculated using the following formula [43]:

(4) $$\begin{array}{c} x=\frac{\pi d^{2}}{4}, \end{array}$$

where *d* = DBH and π = 3.14.

The IVI indicates the importance of species in the system and calculated with three components as follows [41]:

(5) $$\begin{array}{c} \text{RD}=\frac{\text{Number of individuals of species }}{\text{Total number of individuals }}*100, \end{array}$$

(6) $$\begin{array}{c} \text{RDO}=\frac{\text{Dominance of species }}{\text{Total dominance of all species}}*100, \end{array}$$

(7) $$\begin{array}{c} \text{RF}=\frac{\text{Frequency of species }}{\text{Total frequency of all species }}*100. \end{array}$$

The IVI of each woody species is as follows:

(8) $$\begin{array}{c} \text{IVI}=\text{RD}+{\text{RD}}_{O}+\text{RF}. \end{array}$$

### 2.8. Regeneration Status Analysis

The regeneration status of trees and shrubs was examined through comparison of woody plant species. The regeneration status of tree species in the study area was determined by comparing seedlings and saplings of the respective adult species based on density. We classified the life stages (trees, sapling, and seedlings) for these individuals [44]. Seedlings were the under growths of woody species with the height of less than 1 m, trees were single-stemmed individuals with a height of more than 2 m, and saplings were those in between seedlings and trees/shrubs with a height of 1–2 m. The regeneration status was categorized as follows: (1) “Good” regeneration, if seedlings > or < saplings > adults; (2) “Fair” regeneration, if seedlings > or ≤ saplings ≤ adults; (3) “Poor” regeneration, if a species survives only in sapling stage, but no seedlings (though saplings may be < or ≥ adults); (4)“None” or not regenerating, if it is absent both in sapling and seedling stages, but only found in adults; and (5) “New”, if a species has no adults, but only saplings and/or seedling stages. Histograms were drawn using Microsoft Excel Software. The concept of the study data sources, data analysis, and outputs can be summarized as follows in Figure 5.

## 3. Results and Discussions

### 3.1. Woody Plant Species Composition

A total of 28 woody species belonging to 23 genera and 17 families were recorded and identified in the 52 quadrats of the Basso Subwatershed (Table 1). The highest number of species was recorded for Fabaceae family that accounted for 21.4% followed by Anacardiaceae and Combretaceae families that accounts 10.7% each. Families Capparidaceae, Sapindaceae, and Tiliaceae comprised 7.1% species each. The remaining 10 families were represented by one species (Table 1).

The collected species were composed of 50% trees and 50% shrubs (Table 2). The dominance of Fabaceae in the present study coincides with previous woodland vegetation studies from different parts of Ethiopia [4, 21, 34, 45]. The reason for the dominance of Fabaceae might be related to the wider ecological adaptation capability of these leguminous species [46] and excellent dispersal capacities [47]. Moreover, Fabaceae is the dominant family in vegetation ecosystems. This is due to their ability to form symbiotic relationships with nitrogen-fixing bacteria, its adaptability to diverse climate and soil conditions [48]. However, the number of woody species composition of Basso Subwatershed was smaller than in other studies undertaken in Hirmi woodland vegetation in Tigray Region (171 species) (Girmay et al.) [4], Sire Beggo in Gololcha District, Eastern Ethiopia (185 species) (Dibaba et al.) [34], and Metema Area, Amhara National Regional State (87 species) [37]. As seen in Table 2, the nearby communities use woodland of the Basso Subwatershed for a variety of ethnobotanical purposes, including as building materials, animal feed, medicinal properties, and more.

### 3.2. Plant Community Classification

The vegetation classification was done by using the percent cover abundance value data to estimate each species included in the analysis. Optimum number of plant community types was determined by the elbow method (Figure 3) and clustered using Mantel test of distance matrix between species abundance and environmental variables in *R* software, respectively.

**Figure 6:**
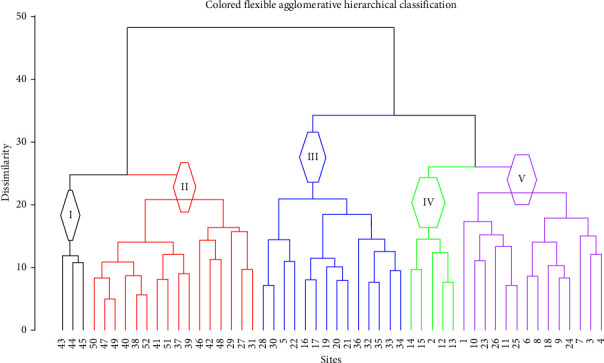
Dendrogram showing plant community type of the study area.

The result of the agglomerative hierarchical cluster analysis program shows that five plant community types were identified (Figure 6).

The identified plant community types in the woodland of subwatershed were *Senegalia mellifera* subsp. *detinens—Acalypha fruticosa* community type I, *Terminalia brownie—Vachellia abyssinica* community type II, *Combretum molle—Vachellia brevispica* community type III, *Terminalia brownie—Acalypha fruticosa* community type IV, and *Euclea divinorum—Dodonaea angustifolia* community type V (Figure 6). These identified community types were higher than those in previous studies conducted in different parts of the country [4, 37, 45]. However, equal community types were encountered in a study undertaken in Gololcha District, Eastern Ethiopia [32].

#### 3.2.1. *Senegalia mellifera subsp. detinens*: *Acalypha fruticosa* Community Type I

This community type was recorded at elevations of 1235 and 1317 m.a.s.l. in 8 quadrats, and it consisted of 24 species (Table 3). The dominant species to this community were *Senegalia mellifera* subsp. *detinens and Acalypha fruticosa*. The other woody species associated with this community type include *Acacia abyssinica*, *Vachellia ehrenbergiana, Cadaba farinosa, Rhus natalensis, Capparis cartilaginea, Acacia brevispica, Combretum molle, Grewia flavescens, Euclea divinorum, Capparis spinosa, Dichrostachys cinerea, Erythrina abyssinica, Ficus vasta, Rhus vulgaris, Capparis tomentosa, Ziziphus mucronata, Balanites aegyptiaca,* and *Sclerocarya birrea* (Figure 6).

#### 3.2.2. *Terminalia brownie*: *Vachellia abyssinica* Community Type II

This community was located in the altitudinal range between 1256 and 1287 m.a.s.l and recorded in 8 quadrats. The dominant species to this community were *Terminalia brownie* and *Vachellia abyssinica*. The other woody species associated with this community type include *Vachellia ehrenbergiana, Commiphora africana, Pappea capensis, Capparis cartilaginea, Balanites aegyptiaca, Rhus natalensis, Dodonaea angustifolia, Sclerocarya birrea,* and *Ziziphus mucronata.*

#### 3.2.3. *Combretum molle*: *Acacia brevispica* Community Type III

*Combretum molle—Acacia brevispica* Community type recorded in 10 quadrats located in the altitudinal range between 1266 m.a.s.l. and 1353 m.a.s.l (Table 3). The dominant species to this community were *Combretum molle* and *Acacia brevispica*. The other woody species associated with this community type include *Terminalia brownie, Cadaba farinose, Rhus natalensis, Senegalia mellifera* subsp. *detinens, Capparis cartilaginea, Cordia monoica, Euclea divinorum, Grewia flavescens, Rhus vulgaris, Balanites aegyptiaca, Vachellia ehrenbergiana, Acalypha fruticosa, Commiphora africana, Dodonaea angustifolia, Vachellia abyssinica, Pappea capensis, Sclerocarya birrea,* and *Ziziphus mucronata.*

#### 3.2.4. *Terminalia brownie*: *Acalypha fruticosa* Community Type IV

This community is located in the altitudinal range between 1218 and 1300 m.a.s.l and consisted of 10 quadrats (Table 3). The dominant species to this community were *Terminalia brownie* and *Acalypha fruticosa.* The other woody species associated with this community type include *Acacia abyssinica, Commiphora africana, Pappea capensis, Senegalia mellifera* subsp. *detinens, Acacia brevispica, Euclea divinorum, Rhus natalensis, Rhus vulgaris, Acacia ehrenbergiana, Dodonaea angustifolia Grewia flavescens, Combretum molle, Carissa edulis, Capparis cartilaginea, Balanites aegyptiaca, Sclerocarya birrea,* and *Ziziphus mucronata.*

#### 3.2.5. *Euclea divinorum*: *Dodonaea angustifolia* Community Type V

This community type was consisted of 16 quadrats at altitudinal range of 1218 and 1336 m.a.s.l (Table 3). The dominant species to this community were *Euclea divinorum* and *Dodonaea angustifolia.* The other woody species associated with this community type include *Senegalia mellifera* subsp. *detinens, Pappea capensis, Vachellia ehrenbergiana, Rhus natalensis, Dichrostachys cinerea, Terminalia brownie, Combretum molle, Olea europaea, Capparis cartilaginea, Carissa edulis, Balanites aegyptiaca,* and *Ziziphus mucronata.*

Identifying plant communities in any given vegetation is very helpful in understanding biodiversity proxies for sustainable management of these resources and to identify as well environmental gradients that structure plant communities [49], and ecologically sensitive areas [50]. The plant communities in the present study can be categorized under the Somalia-Masai *Acacia-Commiphora* deciduous bushland and thicket [51]. Further confirmation of the vegetation type of the study area was given by Friis et al. [52], revealing that it represents *Acacia-Commiphora* woodland and *Combretum-Terminalia* woodland and wooded grassland. The variation in the nature of plant communities might be caused by differences in ecological characteristics (physiographic nature of sites, mountain, valley, and slopes; Assefa et al.) [50], species diversity and regeneration status of species [47], and disturbance level [50, 53–55]. Barkhadle et al. [56] and Hester et al. [57] also revealed that the type of grazing regimes had a great effect in determining plant cover in Shabelle region, Southern Somalia, and in shaping submontane plant communities in Scottish uplands, respectively. Changes in woodland cover were also ascribed to the influence of such management practices like felling activities [57]. However, in some other studies, mixed results were obtained. There were noticed no significant effects in community structure as a result of grazing removal nor initiated a trajectory of development toward keystone species dominance [58].

### 3.3. Plant Species Diversity and Community Types

Understanding why some communities have high species may not only be a matter of verifying ecological theories, but also had direct practical implications for the conservation planning and management [59]. Based on this assumption, the results of Shannon–Wiener diversity index and evenness for the five communities show that plant community type one had the highest number of species richness (33) and Shannon diversity index 2.88 followed by community type three species richness (32) and Shannon diversity index of 2.82 (Table 4).

Community type 4 followed by community type 5 has comparatively the least species richness value (16), but this community has the highest Shannon evenness value (0.90) and community four had the least diversity and highest similar species (the lowest evenness of species) when compared with the other community types (2.46). As a result, the Shannon–Wiener diversity index of this study was found to be lower than that of previous studies conducted by Wale et al. [21], Dibaba et al. [34], and Girmay et al. [4]. However, higher mean diversity values were reported as compared to Birhane et al. [45] findings in the Tigray region, northern Ethiopia. Similarly, the richness and evenness results of this study were the lowest as compared to other studies done in northwestern Ethiopia (74, 0.63) [21], eastern Ethiopia (96, 0.90) [34], and northern Ethiopia (100, 0.95) [4], respectively.

In the identified plant communities, however, there was moderate diversity and evenness showing more or less even representation of individuals of all species in the sampled plots. Thus, the lowest and highest value of Shannon diversity index and evenness of the study area occur between 2.46–2.88 and 0.78–0.90, respectively. The smallest species richness observed in community type II could be because the community is influenced by anthropogenic impact, as was also pointed out by Didita et al. [60] in the woodland vegetation around Dello Menna, Southeast Ethiopia. Species evenness shows the relative proportional abundance of a species in plots (Table 3). According to Boze and Maryo [61], when there is a high evenness value in a forest, the location of the conservation site might not be important as compared to the forest with a low evenness value.

### 3.4. Sorensen Similarity Among Plant Community Types

Based on SSC, the present study revealed that overall similarity coefficient ranges between 0.34 and 0.62 among all communities. The highest similarity was observed between community II and community III (0.62), while the least similarity was observed between community III and community V (0.34) (Table 5). Wale et al. [21] conducted a comparative study in the Metema Area, except Brihane et al. [44] findings which did show higher similarity index (89%) in northern Ethiopia, and other studies conducted on same vegetation type in Eastern Ethiopia (33%) [34] and northwestern Ethiopia (49%). Wale et al. [21] did reflect similar values. This implies that the woody plant species from the above-compared study areas exhibited intermediate similarity in the current study results. The highest similarity between those communities (II and III) might be attributed to the geographical proximity between some sample quadrats, the closer similarity in altitudinal ranges [62]. While the lower similarity may be due to slope differences, degree of human impact (anthropogenic) action variation, climatic conditions, and species growth ecological requirement variation [62].

### 3.5. Effects of Selected Anthropogenic and Physiographic Variables on Vegetation Patterns

Based on the idea of the individualistic continuum concept of vegetation, the pattern of assemblage of species might have been influenced by complex gradients [49]. Among those different factors, slope, disturbance, and grazing intensity did reflect significant effects on woody vegetation patterns in the Basso Subwatershed (*p* < 0.01) (Table 6; Figure 4). However, altitude, aspect, and human impacts did not show significant effects on woody vegetation patterns in the Basso Subwatershed (*p* > 0.01). Within the above figure of CCA result, the numbers represent different quadrats that are significantly impacted by selected different environmental factors such as slope, disturbance, and grazing intensity. Wana et al. [53], Mirdavoodi et al. [54], and Assefa et al. [50] also reported the influence of slope and ecological disturbance regimes on the distribution of plant species/the relative abundance of plant functional types. As slope did have effects on disturbance, with increasing slope grazing pressure was reduced [54]. For instance, higher ecological disturbances through grazing may have resulted in new plant community types that are susceptible and fragile to change [49]. The results of the present study reveal that integrating environmental heterogeneity and human disturbance with topographic variability opens the opportunity to easily understand the existence of large variation in tree community attributes in woodland vegetation [54].

### 3.6. Vegetation Structure of Woody Species

**Figure 7:**
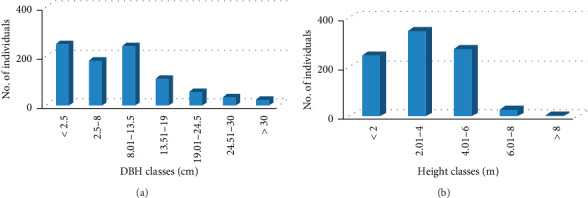
(a and b) Entire woody species at different diameter and height classes.

Vegetation structures of woody species could have vital importance to apply the strategies of conservation, sustainable use, and management [63]. All woody plant species in the study area were classified into seven DBH classes as 1 ≤ 2.5 cm; 2 = 2.5–8 cm; 3 = 8.01–13.5 cm; 4 = 13.51–19 cm; 5 = 19.01–24.5 cm; 6 = 24.51–30 cm; and 7 ≥ 30 cm) and tree height was classified into five height classes (1 ≤ 2 m; 2 = 2.01–4 m; 3 = 4.01–6 m; 4 = 6.01–8 m; and 5 ≥ 8 m), respectively. Similar abundance distribution of woody species in each woody species structures was observed in northwestern Ethiopia [21] (Figures 7(a) and 7(b)).

The present result did show that variations existed in diameter and height classes among the vegetation species (Figures 7(a) and 7(b)). Abundance of species was very high at lower and medium diameter classes and gradually decreased to the higher DBH classes except for a slight decline in the second DBH class (Figure 7(a)). Good abundance of diameter class was found in third diameter class. This general pattern represents an inverted J-shaped curve, and it suggests good reproduction and recruitment capability. Such general inverted J-shaped pattern of normal population structure where the majority of the species exhibited also noted by Didita et al. [60].

**Figure 8:**
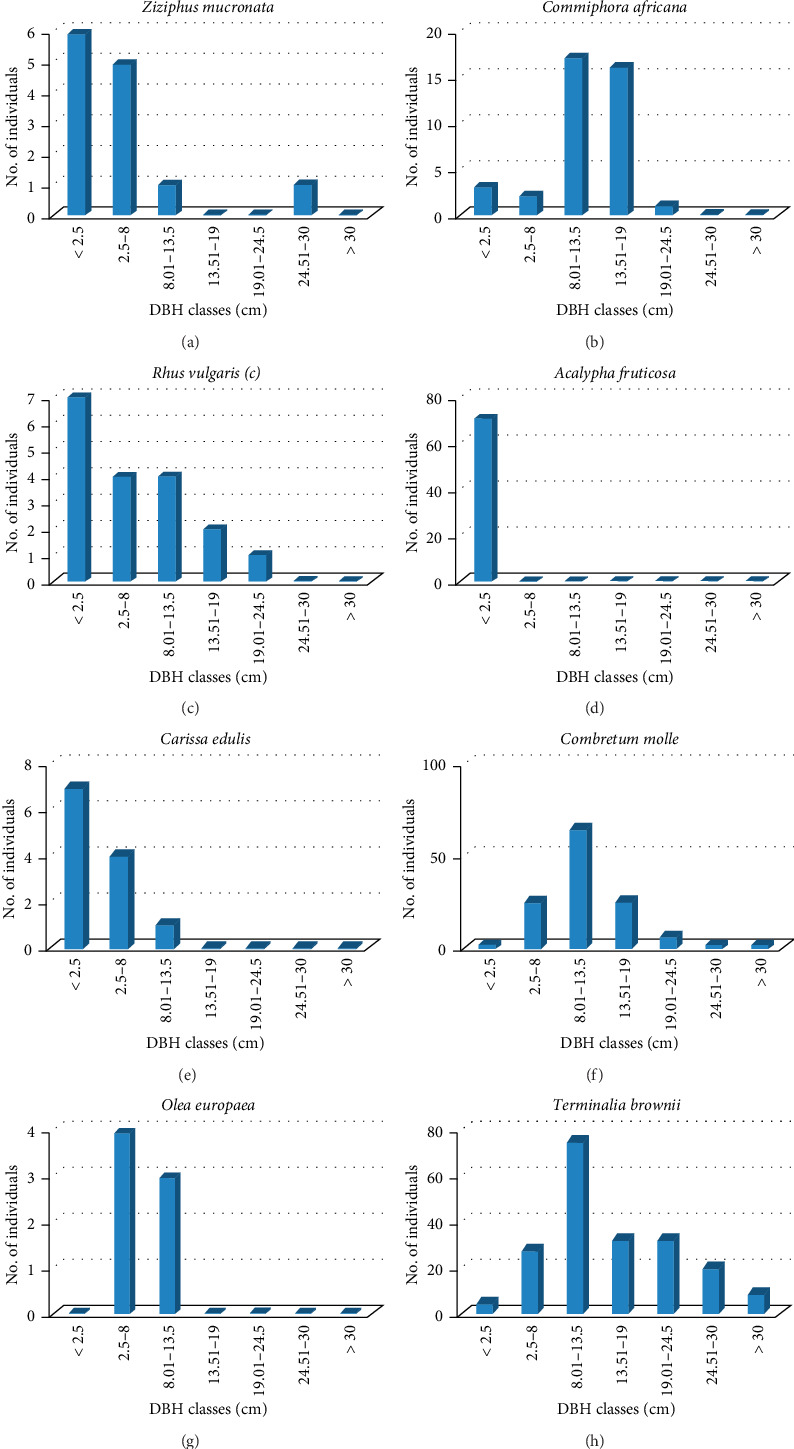
(a–h) DBH class distributions of individual woody species in the Basso Subwatershed. (a) *Ziziphus mucronata,* (b) *Commiphora africana,* (c) *Rhus vulgaris,* (d) *Acalypha fruticosa,* (e) *Carissa edulis,* (f) *Combretum molle,* (g) *Olea europaea,* (h) *Terminalia brownii.*

Similarly, plant population structures were observed in the distribution pattern of the height classes (Figure 8(b)). The general tree height class abundance distribution was higher in the lower height classes and decreasing density was noticed with increase in height classes except for a slight decline in the first height classes (Figure 8(b)). This pattern shows fair population dynamics of the vegetation under the study area. The overall DBH and height class distribution of woody species density in the watershed showed slightly inverted J-shaped structure. Comparable findings were reported from Tigray Region, Northern Ethiopia (Girmay et al.), [4] and Oromia Region, Ethiopia [17].

**Figure 9:**
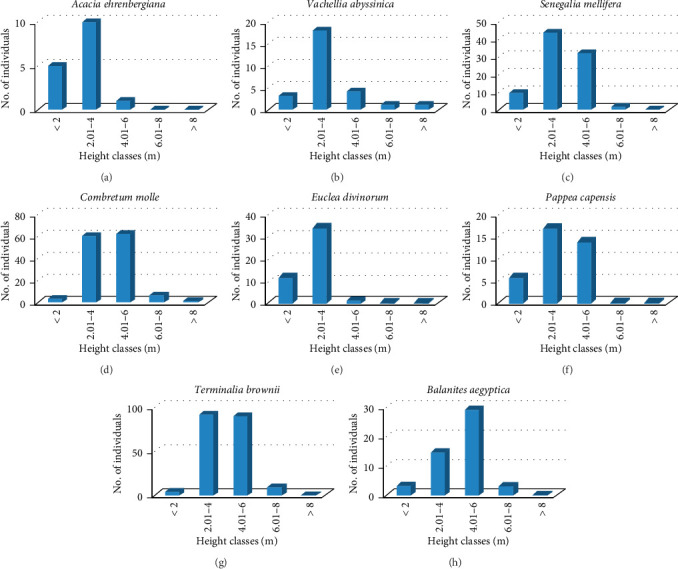
(a–h) Height class distributions of woody species in the Basso Subwatershed. (a) *Acacia ehrenbergiana*, (b) *Vachellia abyssinica*, (c) *Senegalia mellifera*, (d) *Combretum molle*, (e) *Euclea divinorum*, (f) *Pappea capensis*, (g) *Terminalia brownii*, (h) *Balanites aegyptica*.

The population structure of eight representative woody species in the Basso Subwatershed was separately presented for diameter class description in Figures 9(a), 9(b), 9(c), 9(d), 9(e), 9(f), 9(g), 9(h). The study shows that different plant species display various population structures. Based on the analyses of population structures, the study species encountered in the study area were grouped in to four categories as Types I, II, III, and IV. Type I illustrates the situation where the species' diameter or height size class distribution shows a higher proportion of small trees than large trees and a nearly continuous decline in the number of trees from one size class to the next, where such similar distribution patterns were also noted in reports from different parts of Ethiopia [36, 64]. Species with Type II, in which a higher proportion of species were present in intermediate DBH classes and the trend decreased in lower and higher DBH classes, and Type III, which exhibited characteristics such as periodic, irregular, or discontinuous recruitment, reflect a species whose regeneration is severely limited for some reasons [65]. Type IV pattern consists of individual species concentrated only in the first DBH class but absent in the rest classes.

The results for both diameter classes show that *Carissa edulis* and *Rhus vulgaris* were the species grouped as Type I (Figures 8(c) and 8(e)). It was an inverted J-shaped, in which the highest number of individuals was present in lower DBH classes and a nearly continuous decline in the number of trees from one size class to the next. *Commiphora africana, Combretum molle*, and *Terminalia brownii* were grouped as Type II (Figures 8(b), 8(f), 8(h)). Type II was bell-shaped pattern in which a higher proportion of species were present in intermediate DBH classes and the trend decreased in lower and higher DBH classes. *Ziziphus mucronata* and *Olea europaea* as Type III (Figures 8(a) and 8(g)). Type III shows irregular or discontinuous distribution pattern over diameter classes. Such irregular distribution can also be interpreted as broken J-shaped or bell-shaped structure, which was also observed in the woodland vegetation around Dello Menna, Southeast Ethiopia [60]. Some DBH classes had small number of individuals, while other DBH classes had large number of individuals and even some were missed. *Acalypha fruticosa* pattern consists of individual species concentrated only in the first DBH class but absent in the rest of the classes and represented as Type IV (Figure 7(b)). For species like *Acalypha fruticosa,* the reason might be its maximum growth in diameter lies no more than DBH class 1 (< 2.5 cm) and anthropogenic impact due to its importance for different sociological purpose (personal observation).

Additionally, height classes were used to display the population patterns of eight chosen woody species in the Basso Subwatershed (Figures 9(a), 9(b), 9(c), 9(d), 9(e), 9(f), 9(g), 9(h)). The results show that *Vachellia ehrenbergiana*, *Euclea divinorum,* and *Pappea capensis* were categorized in similar patterns of population distributions in the subwatershed. In this pattern, height classes were less abundance of individuals in the first lower height classes followed by high abundance of individuals in the second and third height classes and finally missed the other higher height classes (Figures 9(a), 9(e), 9(f)). It might be due to the observed multiple disturbance factors (cutting, grazing, fire, and charcoal production signs). The species population pattern of *Vachellia abyssinica, Senegalia mellifera* subsp. *detinens*, *Combretum molle*, *Terminalia brownie*, and *Balanites aegyptiaca* have similar patterns (Figures 9(b), 9(c), 9(d), 9(g), 9(h)). In this pattern, the lowest height classes have empty or lower densities followed by increase in the number of individuals toward the middle classes and then a progressive decrease toward the higher height classes and finally absent. Species with such a distribution pattern indicate a poor reproduction which might be associated with different factors that inhibit reproduction, or the presence of only few seed-bearing individuals. The cumulative diameter and height class results show different patterns of population structure revealed a high variation among species populations and an indication of limited mother plants for future healthy regeneration of the species in the watershed, as revealed by Temesgen and Warkineh [28] and Wale et al. [21].

### 3.7. Frequency of Woody Species

The result of RF value in the present study shows that *Terminalia brownii* was the most frequent species with the frequency value of 80.8% followed by *Acalypha fruticosa* (75%), *Senegalia mellifera* subsp. *detinens* (71.2%), *Rhus natalensis* (69.2%), *Combretum molle* (63.2%), and *Acacia brevispica* (53.8%) of the total quadrats sampled (Table 7). On the other hand, the less frequent woody species based on the descending order were *Grewia bicolor* and *Dichrostachys cinerea* (7.7%); *Grewia flavescens* (5.8%); *Cordia monoica, Maytenus senegalensis*, and *Olea europaea* (3.8%); and *Capparis tomentosa, Ceratonia siliqua*, and *Ficus vasta* (1.9%). A complete list of frequency of woody species in the Basso Subwatershed is given in Table 7.

**Figure 10:**
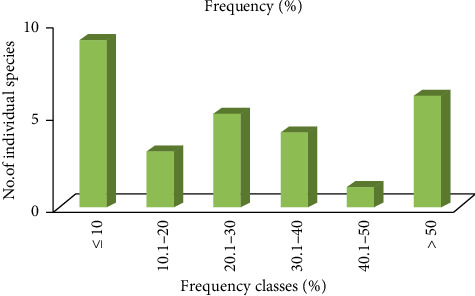
Frequency distribution of entire woody species in frequency classes.

Based on the percentage frequency values, the woody plant species were classified into six frequency classes as (1) < 10, (2) 10.1–20, (3) 20.1–30, (4) 30.1–40, (5) 40.1–50 and > 50, which is expressed in percentage. The present study revealed high percentage of number of species were 9 (32.14%) in lower frequency classes and relatively low percentage of number of species were 6 (21.43%) in the higher frequency classes. However, the only least percentage of number of specie was *Euclea divinorum* 1 (6.6%) in the higher frequency classes (Figure 10). Thus, the result shows the existence of high degree of floristic heterogeneity in the Basso Subwatershed.

According to the previous studies done by Wale et al. [21], Dibaba et al. [34], Girmay et al. [4], and Birhane et al. [45], the most frequently recorded species were *Sterculia setigera*, *Acokanthera schimperi*, *Anogeissus leiocarpa*, and *Senegalia senegal var. rostrata* in each study area, respectively, whereas in this study, *Terminalia brownii* was the most frequently recorded species (80.8%).

### 3.8. Density of Woody Species

The total mean densities of all woody species as calculated from the data taken from 52 sample quadrats of the study area were 5745 stems per hectare (Table 4), which is much higher than studies conducted in Tigray Region, Northern Ethiopia (528.4 ha^−1^) [4] and Sire Beggo in Gololcha District, Eastern Ethiopia (1845 ha^−1^) [34]. The largest density of eight species in the study area was *Acalypha fruticosa* (2763.94 stems ha^−1^), *Terminalia brownii* (660.1 stems ha^−1^), *Vachellia brevispica* (523 stems ha^−1^), *Rhus natalensis* (329 stems ha^−1^), *Dodonaea angustifolia* (306 stems ha^−1^), *Euclea divinorum* (258 stems ha^−1^), *Acacia mellifera* (173 stems ha^−1^), and *Combretum molle* (123 stems ha^−1^) (Table 7).

### 3.9. Basal Area of Woody Species

The total basal area of the Basso Subwatershed woody species calculated from DBH data was 12.8 m^2^·ha^−1^ (Table 7). More than 87% (11.18 m^2^·ha^−1^) of the total basal area was contributed by seven large-sized tree species were *Terminalia brownii*, *Combretum molle*, *Balanites aegyptiaca*, *Sclerocarya birrea, Pappea capensis, Senegalia mellifera* subsp. *detinens*, and *Commiphora africana* (Table 7). Basal area provides an improved measure of the relative importance of species than simple stem count. Thus, species with the largest basal area can be regarded as a vital species or dominant [61]. Accordingly, in the current study, *Terminalia brownii* 4.7(36.7%) m^2^·ha^−1^ followed by *Combretum molle* 2.35 (18.4%) m^2^·ha^−1^, *Balanites aegyptiaca* 1.05 (8.2%) m^2^·ha^−1^, and *Sclerocarya birrea* 1.04 (8.1%) m^2^·ha^−1^ have higher basal area and canopy cover, which are taken as dominant species of the Basso Subwatershed woody species (Table 7).

The highest basal area from individual tree species in the study was contributed by *Terminalia brownii* (4.7 m^2^·ha^−1^), while the highest density was *Acalypha fruticosa* (2763.94 individuals ha^−1^). In this study, basal area analysis across individual species revealed that there was high domination by very few or small woody species. This indicates that species with the highest basal area do not necessarily have the highest density, and vice versa. It was indicated that size difference between species is common in natural vegetation [29]. In the current study, total basal area was lower than that conducted by Dibaba et al. [34] in Sire Beggo, Gololcha District, Eastern Ethiopia (19.3 m^2^·ha^−1^), and by Girmay et al. [4] in Hirmi woodland in Tigray Region (14 m^2^·ha^−1^). The least basal area noted in the present subwatershed may be due to the presence of individual tree species in the Basso Subwatershed with relatively lower DBH than in the woodland compared. This also indicates that the Basso Subwatershed woodland is found relatively lower stage of development than Sire Beggo and Hirmi woodlands.

### 3.10. IVI of Woody Species

Curtis and McIntosh [63] pointed out that IVI gives a more realistic figure of dominance from the structural point of view. It is important to compare the ecological significance of species [64] and good index for vegetation characteristics and ranking species for management and conservation priority. The IVI of woody species in the study area ranged from 0.3% to 59% (Table 3). The first top ten woody species that contributed 81% of the IVI in decreasing order were *Acalypha fruticosa, Terminalia brownii*, *Combretum molle*, *Senegalia mellifera* subsp. *detinens, Acacia brevispica, Rhus natalensis, Balanites aegyptiaca, Sclerocarya birrea, Pappea capensis,* and *Euclea divinorum.* These woody species were abundant, frequent, dominant, and ecologically most significant in the study area. The remaining percentages were shared among the other 18 species in Table 7.

Significant conservation effort is often required for species with lower important value indices, whereas monitoring and management are necessary for species with higher IVI [61]. In the current study, the highest IVI was contributed by *Acalypha fruticosa* (58.8) and *Terminalia brownii* (58) value and the least was contributed by *Ficus vasta* (0.27). Comparative study in Tigray Region, Northern Ethiopia [4], IVI was reported that *Anogeissus leiocarpa* (20.55) and *Vachellia nilotica* (0.35) to have the highest and lowest value proportion, respectively. In this study, *Acalypha fruticosa* and *Terminalia brownii* with high IVI value depict that the species sociological structure in the community has high and requires monitoring and management, whereas *Ficus vasta, Capparis tomentosa*, and *Ceratonia siliqua* have least IVI values that require high conservation priority (Table 7). Accordingly, the previous study which carried out by Wale et al. [21], Dibaba et al. [34], and Girmay et al. [4] states that IVI of the top dominant woody species was accounted to 63%, 59.6%, and 37.78%, respectively, but in this study, the first top ten woody species contributed to 81% of IVI value. Accordingly, based on IVI result, it can be conclude that the Basso Subwatershed woodland had better ecological significance of species than previous compared woodlands.

### 3.11. Regeneration Status of Woody Species in the Basso Subwatershed

**Figure 11:**
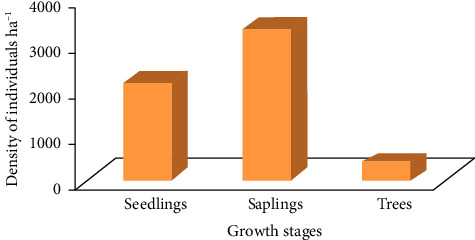
Regeneration status of the entire woody species.

Assessments of seedling and sapling on the basis of the composition, distribution, and densities were effective criteria for successful prioritize conservation measurement of the woodland resources and to determine the regeneration status [4]. Out of the total identified woody species (Figure 11), *Acalypha fruticosa* showed the highest density of seedling and sapling, with 1088 ha^−1^ and 1675 ha^−1^, respectively, while *Grewia flavescens* had the lowest, with seedling and sapling densities of 1.4 ha^−1^ and 0.5 ha^−1^, respectively, in the study area (Table 8). The diversity and structure of the forest may suffer long-term consequences if the dominating species exhibit inadequate regeneration. A dominant species may experience less regeneration, which could result in a drop in its total population and open the door for other species to gain prominence. Thus, species with poor regeneration require management and protection in the woodland, and those with stronger regeneration in this study recommend low management [65].

According to Dhaulkhandi et al. [66], a given forest had good regeneration if seedling is greater than sapling and mature tree/adult (seedling density > sapling density > mature tree/adults); fair regeneration if seedling > or ≤ sapling ≤ mature tree; poor regeneration if seedling < sapling ≥ or ≤ mature tree; and no regeneration if species are represented only by adult/mature trees. From the analysis, the total density of seedling, sapling, and adult trees/shrubs was 2131.3 ha^−1^ (37.1%), 3298.6 ha^−1^ (57.4%), and 318.7 ha^−1^ (5.5%), respectively (Figure 10). The result shows the distribution of sapling was greater than seedlings and mature individuals, indicating that the studied vegetation of the Basso Subwatershed had categorized as fair regeneration status.

**Figure 12:**
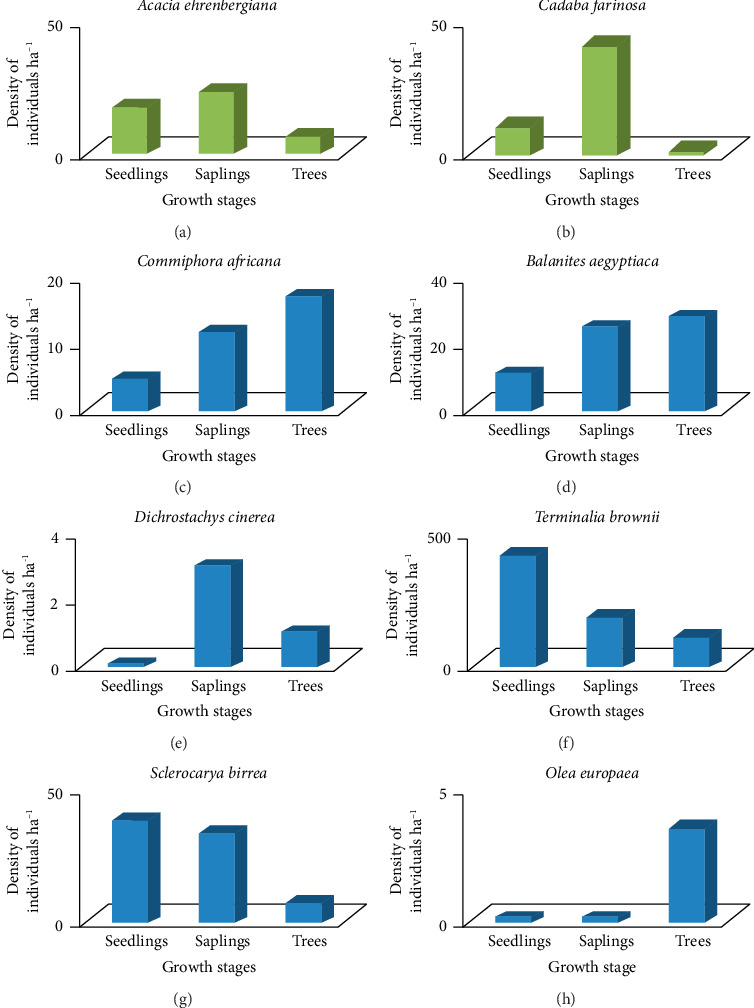
(a–h) Regeneration statuses of selected woody species in the study area. (a) *Acacia ehrenbergiana*, (b) *Cadaba farinosa*, (c) *Commiphora africana,* (d) *Balanites aegyptiaca,* (e) *Dichrostachys cinerea,* (f) *Terminalia brownii,* (g) *Sclerocarya birrea,* (h) *Olea europaea*.

Based on the criteria of Dhaulkhandi et al. [66], the randomly selected woody species of the Basso Subwatershed were generally categorized under four regeneration levels (Figure 12). The frequency histograms of these species as an individual vegetation of the watershed revealed that, good regeneration, the density value of populations increased from seedlings to saplings, and then, trees *Terminalia brownii* and *Sclerocarya birrea* (Figures 12(f) and 12(g)). These two species showed relatively normal trends of regeneration status of an inverted “J” shape from the total of 28 identified woody species in the study area [28]. Fair regeneration, *Vachellia ehrenbergiana*, *Commiphora africana*, and *Balanites aegyptiaca* (Figures 12(a), 12(c), 12(d)), poor regeneration *Dichrostachys cinerea* and *Cadaba farinosa*Figure 11 and no regeneration *Olea europaea* (Figure 12(h)). The tree species, which had no seedlings and saplings, exhibited discontinuous population structures [28].

The “good” and “fair” regenerating categories which constituted around 15 (53.57%) of the woody plant species in the Basso Subwatershed have many important and useful tree species. These species were *Combretum molle*, *Balanites aegyptiaca*, *Pappea capensis, Senegalia mellifera* subsp. *detinens, Commiphora africana, Vachellia abyssinica, Vachellia ehrenbergiana, Cadaba farinosa, Capparis cartilaginea, Carissa edulis, Euclea divinorum, Grewia flavescens, and Rhus vulgaris, Terminalia brownii*, and *Sclerocarya birrea* (Table 9). The assessment of regeneration status based on seedling and sapling count also showed that a significant proportion of woody species were fair regenerating, implying that they are more or less under threat [29]. It is therefore imperative to develop and implement effective conservation measures to save the woody species of the subwatershed.

Based on total regeneration status of woody plant species, comparable mean values were also reported for total density of seedlings (2750 ha^−1^), saplings (3025 ha^−1^), and mature tree species (528.4 ha^−1^) in Tigray Region, at Hirmi woodland [4], and similar patterns in Gololcha District, Eastern Ethiopia (1108 ha^−1^, 396 ha^−1^, and 60 ha^−1^, respectively, Dibaba et al. [34]), and in North-Western Tigray (73.3 ha^−1^, 102.8 ha^−1^, 656.7 ha^−1^, respectively, Birhane et al. [45]). Based on the comparison of seedlings, saplings, and mature tree species density patterns, it can be concluded that the Basso Subwatershed woodland was fair regeneration status.

## 4. Conclusion

A total of 28 plant species that belong to 17 families and 23 genera were recorded, and it was found that Basso Subwatershed had not rich plant species diversity compared to other similar woodlands of the country. In terms of family, Fabaceae was found to be the most dominant family followed by *Combretaceae* and *Anacardiaceae*. It found that vegetation was grouped into five plant community types. There was not a big difference in Shannon diversity index, species richness, and evenness among the five communities.

*Acalypha fruticosa*, *Terminalia brownii*, *Combretum molle*, and *Balanites aegyptiaca* were the most dominant woody species in the subwatershed. Moreover, *Vachellia brevispica*, *Senegalia mellifera* subsp. *detinens*, *Acalypha fruticosa*, *Combretum molle*, *Rhus natalensis*, and *Terminalia brownii* were the most frequent woody plant species. The cumulative diameter and height class frequency distribution of population structure of most common species of trees and shrubs revealed different patterns of population structure, addressing a high variation among species population regenerations within the watershed and shows an indication of fair regeneration. The analysis of population structure shows that woody species like *Olea europaea*, *Ficus vasta*, *Capparis tomentosa*, *Cordia monoica*, and *Ceratonia siliqua* have none/limited to regeneration potential and calls the need for farther research and conservation priority. It also indicates that there is selective removal of certain woody species in the woodland.

The analysis of frequency classes for woody species revealed higher percentage of species number of individuals in the lower frequency classes which is evident for the floristic heterogeneity of the woodland. The IVI shows that 81% of the species in the woodland were woody plants, with the most common species varieties, namely, *Acalypha fruticosa*, *Terminalia brownii*, *Combretum molle*, *Senegalia mellifera* subsp. *detinens*, *Vachellia brevispica*, *Rhus natalensis*, *Balanites aegyptiaca*, *Sclerocarya birrea, Pappea capensis*, and *Euclea divinorum.* Of these, *Acalypha fruticosa*, *Terminalia brownii*, and *Combretum molle* are the most dominant tree species in the Basso Subwatershed.

A great fraction of woody species exhibit fair regeneration and are basically in danger of deterioration. To encourage regeneration in species with low IVI values or poor regeneration status, research and/or development action is required. We may implement techniques for preserving water, soil restoration, and biological and physical integration with subwatershed resource management to expedite the process. More investigation or actions are required to reduce human impacts that are heavily reliant on the woodland vegetation. In addition to investigate the association between community types and physiographic factors and the association between community types, long-term monitoring, and impact of specific human activities, detailed soil analysis on plant distribution should be investigated in the future.

## Figures and Tables

**Table 1:** Plant families, number of genera, and species of woody plant species.

No	Family	Number of genera	(%)	Number of species	(%)
1	Fabaceae	6	12.20	6	21.43
2	Euphorbiaceae	3	6.12	1	3.57
3	Balanitaceae	1	2.04	1	3.57
4	Capparidaceae	3	6.12	2	7.14
5	Combretaceae	2	4.08	3	10.71
6	Apocynaceae	1	2.04	1	3.57
7	Burseraceae	19	38.80	1	3.57
8	Boraginaceae	2	4.08	1	3.57
9	Sapindaceae	3	6.12	2	7.14
10	Ebenaceae	1	2.04	1	3.57
11	Moraceae	1	2.04	1	3.57
12	Tiliaceae	2	4.08	2	7.14
13	Celastraceae	1	2.04	1	3.57
14	Oleaceae	2	4.08	1	3.57
15	Anacardiaceae	1	2.04	3	10.7
16	Rhamnaceae	1	2.04	1	3.57

Total		49	100.00	28	100.00

**Table 2:** List of woody plant species, families, and their growth forms.

No	Scientific name	Family	Plant habit	Local use
1	*Vachellia abyssinica* Hochst.	Fabaceae	T	Firewood, charcoal, poles, posts, tool handles, edible gum.
2	*Acacia brevispica* Harms.	Fabaceae	S	firewood, fodder, live fence
3	*Acacia ehrenbergiana* Hayne	Fabaceae	T	Animal feed, edible gum, firewood and charcoal
4	*Acacia mellifera* (Vahl) Benth.	Fabaceae	T	Animal fodder, construction materials
5	*Acalypha fruticosa* Forssk.	Euphorbiaceae	S	Food source and medicinal purpose
6	*Balanites aegyptiaca* (L.) Del.	Balanitaceae	T	Fruit is edible, construction materials
7	*Cadaba farinosa* Forssk.	Capparidaceae	S	Food source and for medicinal purposes
8	*Capparis cartilaginea *Decne.	Combretaceae	S	Traditional medicine
9	*Capparis tomentosa* Lam.	Capparidaceae	S	To treat coughs, fever, and headaches
10	*Carissa edulis* (Forssk.)	Apocynaceae	S	Food and medicine
11	*Ceratonia siliqua* L.	Fabaceae	S	Food and medicine
12	*Combretum molle* R. Br.	Combretaceae	T	Medicine value
13	*Commiphora africana* (A. Rich)	Burseraceae	T	Medicine, food, and handicraft applications
14	*Cordia monoica Roxb.*	Boraginaceae	T	Fruits are eaten
15	*Dichrostachys cinerea* (L.)	Fabaceae	S	Medicine, fodder, and fuel wood
16	*Dodonaea angustifolia* L. f.	Sapindaceae	S	Medicine, firewood, and hedge plant
17	*Euclea divinorum* Hiern	Ebenaceae	S	Medicine, food preservation
18	*Ficus vasta* Forssk.	Moraceae	S	Shade, food
19	*Grewia bicolor* Juss.	Tiliaceae	S	Tool making, food, medicine
20	*Grewia flavescens *Juss.	Tiliaceae	T	Food and traditional medicine
21	*Maytenus senegalensis* (Lam.)	Celastraceae	T	Traditional medicine.
22	*Olea europaea* L.	Oleaceae	T	Furniture, fence posts, medicine
23	*Pappea capensis* Eckl. & Zeyh.	Sapindaceae	T	Fruit is edible, medicine
24	*Rhus natalensis* Krauss	Anacardiaceae	S	Medicinal purposes
25	*Rhus vulgaris* Meikle	Anacardiaceae	T	Toothbrush, medicine
26	*Sclerocarya birrea* (A. Rich.) Hochst.	Anacardiaceae	T	Food
27	*Terminalia brownii* Fresen.	Combretaceae	T	Construction, fuel, and tool handles
28	*Ziziphus mucronata* Willd.	Rhamnaceae	S	Medicine

**Table 3:** Distribution of quadrats of the five plant communities with their altitudinal ranges.

PCT	TQs	List of quadrats	AR (m.a.s.l.)	MS (%)	Aspect	Human activities		
						HI	GI	DF
Community I	8	1, 2, 3, 4.5, 6, 7, 8	1235–1307	22.9	West	Very high	Low	low
Community II	8	9, 10, 11, 12, 13, 14, 24,25	1256–1287	7.5	East	Very high	Low	High
Community III	10	15, 16, 17, 18, 19, 20, 21, 22, 23.52	1266–1353	15.2	South, East	High	Low	High
Community IV	10	26, 30, 31, 36, 37, 38, 39, 40, 47, 51	1218–1300	17.7	East	High	Low	High
Community V	16	27, 28, 29, 32, 33, 34, 35, 41, 42.43, 44, 45, 46, 48,49, 50	1218–1336	20.7	West, East	High	Very low	Medium

*Note:* PCT is community types, TQs are total quadrats, DI is disturbance factor.

Abbreviations: AR, altitude range; GF, grazing intensity; HI, human impact; MS, mean slope.

**Table 4:** Plant species diversity of plant community.

Plant community type	Richness	Shannon diversity index(*H*′)	Shannon evenness index (*J*)
I	33	2.88	0.82
II	16	2.49	0.90
III	32	2.82	0.81
IV	23	2.46	0.78
V	19	2.60	0.88

**Table 5:** Sorenson's similarity index among each community types.

Plant community types	I	II	III	IV	V
I					
II	0.53				
III	0.56	0.62			
IV	0.57	0.56	0.58		
V	0.36	0.41	0.34	0.57	

**Table 6:** ANOVA showing the effect of environmental factors on vegetation patterns.

Environmental factors	Df	Sum of squares	Mean of squares	F model	*R* ^2^	Pr (> F)
Altitude	1	0.32	0.32	1.31	0.02	0.23
Slope	1	0.76	0.76	3.12	0.05	0.004^∗∗^
Aspect	1	0.15	0.15	0.60	0.01	0.808
Human impact	1	0.38	0.38	1.52	0.03	0.12
Grazing	1	0.89	1.89	3.56	0.06	0.002^∗∗^
Disturbance	1	0.67	0.67	2.70	0.05	0.009^∗∗^
Residuals	45	11.19	0.25	0.78		
Total	51	14.37			1.0	

^∗∗^Significant at *p* < 0.01.

**Table 7:** Woody plant structure of Basso Subwatershed, South Ethiopia Regional Government.

Scientific name	BA	F	RF	D	RD	DO	RDO	IVI
*Vachellia abyssinica* Hochst.	0.39	26.9	3.41	91.34	1.59	0.19	3.06	8.1
*Vachellia brevispica* Harms.	0.06	53.8	6.81	523.07	9.11	0.03	0.47	16.4
*Vachellia ehrenbergiana* Hayne	0.07	17.3	2.19	45.67	0.8	0.03	0.55	3.6
*Senegalia mellifera* (Vahl) Benth. subsp. *detinens*	0.65	71.2	9	173.55	3.02	0.31	5.1	17.13
*Acalypha fruticosa* Forssk.	0.15	75	9.49	2763.9	48.11	0.08	1.22	58.8
*Balanites aegyptiaca* (L.)	1.05	36.5	4.62	58.65	1.02	0.5	8.19	13.8
*Cadaba farinosa* Forssk.	0.07	11.5	1.46	48.55	0.85	0.03	0.55	2.85
*Capparis cartilaginea* Decne	0.17	30.8	3.89	51.44	0.9	0.08	1.29	6.1
*Capparis tomentosa* Lam.	0.003	1.9	0.24	1.44	0.03	0	0.02	0.29
*Carissa edulis* (Forssk.)	0.03	21.2	2.68	39.42	0.69	0.02	0.25	3.62
*Ceratonia siliqua* L.	0.002	1.9	0.24	1.44	0.03	0	0.02	0.28
*Combretum molle* R. Br.	2.35	63.5	8.03	123.5	0.15	1.13	18.41	28.6
*Commiphora africana* (A. Rich)	0.59	32.7	4.14	33.65	0.59	0.28	4.61	9.33
*Cordia monoica* Roxb.	0.01	3.8	0.49	1.44	0.03	0	0.08	0.59
*Dichrostachys cinerea* (L.)	0.02	7.7	0.97	3.84	0.07	0.01	0.15	1.2
*Dodonaea viscosa* subsp. *angustifolia* (L.f.) J.G.West	0.03	17.3	2.19	306.7	5.34	0.01	0.19	7.72
*Dodonaea viscosa* subsp. angustifolia (L.f.) J.G.West	0.03	17.3	2.19	306.7	5.34	0.01	0.19	7.72
*Euclea divinorum* Hiern	0.22	40.4	5.11	258.1	4.49	0.11	1.74	11.4
*Ficus vasta* Forssk.	0.002	1.9	0.24	0.48	0.01	0	0.02	0.27
*Grewia bicolor* Juss.	0.02	7.7	0.97	7.21	0.13	0.01	0.19	1.28
*Grewia flavescens *Juss.	0.02	5.8	0.73	3.36	0.06	0.01	0.13	0.92
*Maytenus senegalensis* (Lam.)	0.01	3.8	0.49	2.88	0.05	0.01	0.1	0.64
*Olea europaea* L. subspecies *cuspidata*	0.04	3.8	0.49	3.36	0.06	0.02	0.33	0.9
*Pappea capensis* Eckl. & Zeyh.	0.8	34.6	4.38	45.67	0.9	0.39	6.27	11.44
*Rhus natalensis* Krauss	0.1	69.2	8.76	329.32	5.73	0.04	0.67	15.16
*Rhus vulgaris* Meikle	0.2	23.1	2.92	70.67	1.23	0.08	1.25	5.4
*Sclerocarya birrea* (A.Rich.)	1.04	23.1	2.92	74.52	1.3	0.5	8.13	12.35
*Terminalia brownii* Fresen.	4.7	80.8	10.2	660	11.5	2.24	36.34	58.04
*Ziziphus mucronata* Willd.	0.1	23.1	2.92	21.63	0.38	0.04	0.65	3.95
Total	12.8		100	745.19	100	6.157	100	300

**Table 8:** Number of seedling, sapling, and matured tree of woody plant species.

No	Scientific name	Number of seedlings	Number of saplings	Adult trees	Total
1	*Vachellia abyssinica* Hochst.	72	94	24	190
2	*Vachellia brevispica* Harms.	366	722	0	1088
3	*Vachellia ehrenbergiana* Hayne	36	47	12	95
4	*Senegalia mellifera* subsp. *detinens* (Vahl) Benth.	67	208	79	354
5	*Acalypha fruticosa* Forssk.	2264	3485	0	5749
6	*Balanites aegyptiaca* (L.) Del.	22	52	58	132
7	*Cadaba farinosa* Forssk.	19	81	1	101
8	*Capparis cartilaginea *Decne.	15	85	7	107
9	*Capparis tomentosa* Lam.	0	3	0	3
10	*Carissa edulis* (Forssk.)	14	57	5	76
11	*Ceratonia siliqua* L.	0	3	0	3
12	*Combretum molle* R. Br.	21	107	129	257
13	*Commiphora africana* (A. Rich)	10	24	36	70
14	*Cordia monoica* Roxb.	0	0	3	3
15	*Dichrostachys cinerea* (L.)	0	6	2	8
16	*Dodonaea angustifolia* L. f.	263	375	0	638
17	*Euclea divinorum* Hiern	145	355	37	537
18	*Ficus vasta* Forssk.	0	1	0	1
19	*Grewia bicolor* Juss.	0	11	1	12
20	*Grewia flavescens *Juss.	3	1	3	7
21	*Maytenus senegalensis* (Lam.)	0	5	1	6
22	*Olea europaea* L.	0	0	7	7
23	*Pappea capensis* Eckl. & Zeyh.	27	37	32	96
24	*Rhus natalensis* Krauss	145	540	0	685
25	*Rhus vulgaris* Meikle	29	107	11	147
26	*Sclerocarya birrea* (A. Rich.) Hochst.	79	68	13	160
27	*Terminalia brownii* Fresen.	836	350	190	1376
28	*Ziziphus mucronata* Willd.	0	35	7	42

Total		4433	6859	658	11,950
Total density (ha)		2131.3	3295.6	318.7	5745.2
Total density (%)		37.1	57.4	5.5	100

**Table 9:** Regeneration status of woody plant species in the Basso Subwatershed.

Scientific name	SD	SPD	T/SD	TD	RS
*Vachellia abyssinica* Hochst.	34.6	45.2	11.6	91.3	Fair
*Vachellia brevispica* Harms.	176	347	0	523.1	Poor
*Vachellia ehrenbergiana* Hayne	17.3	23	6	45.7	Fair
*Senegalia mellifera* subsp. *detinens* Vahl Benth.	32.2	100	38.3	170.2	Fair
*Acalypha fruticosa Forssk.*	1088.4	1675	0	2764	Poor
*Balanites aegyptiaca* L. Del.	10.5	25	28.1	63.5	Fair
*Cadaba farinosa Forssk.*	9.1	40	0.5	48.6	Fair
*Capparis cartilaginea Decne*.	7.2	41	3.4	51.4	Fair
*Capparis tomentosa* Lam.	0	5	0	1.44	None
*Carissa edulis* Forssk.	6.7	27.3	2.4	36.5	Fair
*Ceratonia siliqua* L.	0	2	0	1.44	None
*Combretum molle* R. Br.	10	51.4	62.5	123.6	Fair
*Commiphora africana* A. Rich	4.8	12	17.4	33.7	Fair
*Cordia monoica* Roxb.	0	0	1.5	1.44	None
*Dichrostachys cinerea* L.	0	3	1	4	Poor
*Dodonaea angustifolia* L. f.	126.5	180.2	0	307	Poor
*Euclea divinorum* Hiern	69.7	171	18	258.2	Fair
*Ficus vasta* Forssk.	0	0.5	0	0.5	None
*Grewia bicolor* Juss.	0	5.3	0.5	8	Poor
*Grewia flavescens* Juss.	1.4	0.5	1.5	3.4	Fair
*Maytenus senegalensis* Lam.	0	2.4	1.5	3	Poor
*Olea europaea* L.	0	0	3.4	3.4	None
*Pappea capensis* Eckl. & Zeyh.	13	18	15.5	46.2	Fair
*Rhus natalensis* Krauss	70	260	0	329.3	Poor
*Rhus vulgaris* Meikle	14	51.4	5.3	70.7	Fair
*Sclerocarya birrea* (A. Rich.)	38	33	6.3	77	Good
*Terminalia brownii* Fresen.	402	168.2	92	661.5	Good
*Ziziphus mucronata* Willd.	0	17	3.4	20.2	Poor
Total	2131.3	3295.6	318.7	5745.2	
Total density in %	37.1	57.4	5.5	100	

Abbreviations: RS, regeneration status; SD, seedling density; SPD, sapling density; TD, total density (ha^−1^); T/SD, tree/shrubs density.

## Data Availability

All data generated and analyzed during this study are available in the manuscript.
